# Supplementary material for: Assessment of glycemic control, health-related quality of life, and associated factors in type 2 diabetic patients attending a comprehensive specialized hospital in Northwest Ethiopia
Source: PLoS One. 2025 Sep 18;20(9):e0332628. doi: 10.1371/journal.pone.0332628 (PMC12445461; doi:10.1371/journal.pone.0332628)
Supplement: Attachment — Submitted filename: Response to Editor Comments.docx [file pone.0332628.s004.docx]

**Response to Editor Comments**

**Manuscript Title:** Assessment of glycemic control, health-related quality of life, and associated factors in type 2 diabetic patients attending a comprehensive specialized hospital in northwest Ethiopia

**Manuscript Submission Number:** PONE-D-25-24519R1

**Journal:** PLOS ONE

Dear Editor,

We sincerely thank you for your feedback. Your feedback is a valuable input to our efforts to improve the clarity of the sample size calculation and ensure consistency in reporting blood glucose measurements using appropriate units. Moreover, we are so much grateful for your prompt decisions, which are crucial to facilitate a timely final decision on our manuscript.

In response to your comments, we have made some additional revisions. This submission includes previous revisions plus some additional amendments on the sample size calculation section.

Journal Requirements:

1. If the reviewer comments include a recommendation to cite specific previously published works, please review and evaluate these publications to determine whether they are relevant and should be cited. There is no requirement to cite these works unless the editor has indicated otherwise.

No specific previously published work was recommended for citation.

2. Please review your reference list to ensure that it is complete and correct. If you have cited papers that have been retracted, please include the rationale for doing so in the manuscript text, or remove these references and replace them with relevant current references. Any changes to the reference list should be mentioned in the rebuttal letter that accompanies your revised manuscript. If you need to cite a retracted article, indicate the article’s retracted status in the References list and also include a citation and full reference for the retraction notice.

We have checked our reference list. All references are available online and none of them are retracted.

**Additional Editor Comments:**

All reviewer’s comments should be addressed in full. Please use SI units for the glucose measures. The sample size calculation should be revised and better explained.

The paper reports interesting data.

Thank you dear Editor for recognizing that our paper presents interesting data.

**SI units for glucose measurements:**

All blood glucose values (which were expressed in mg/dl) have been converted to SI compatible, specifically millimoles per liter (mmol/L) throughout the manuscript.

These changes have been incorporated in the revised manuscript, and all modifications are highlighted in blue text for ease of visibility.

Although the actual SI unit for blood glucose measurement is mol/m³, glucose concentrations are commonly expressed in millimoles per liter (mmol/L) in biomedical and clinical practices. This unit is widely used in guidelines such as those of the American Diabetes Association, scientific literatures, and books. In response to the reviewer’s comment, we have changed all glucose concentration expressions in the manuscript from **mg/dl** to **mmol/L**. Even though **mmol/L** is not SI unit, it is an **SI-compatible derived unit** and is equivalent to **mol/m³** and used commonly as alternative of mg/dL expressions practically. Mathematically, mmol/L is equal to mol/m³. (10^-3^×mol)/10-^3^m^3^ = mol/m^3^.

Moreover, we would be happy if you could kindly specify the preferred SI unit you would like us to use, so we can make the necessary corrections accordingly.

**References (just to support our reflections)**

1. American Diabetes Association Professional Practice Committee (2024). 6. Glycemic Goals and Hypoglycemia: Standards of Care in Diabetes-2024. Diabetes care, 47(Suppl 1), S111–S125. <https://doi.org/10.2337/dc24-S006>
2. Renard E, Farret A, Kropff J, et al. Day-and-Night Closed-Loop Glucose Control in Patients With Type 1 Diabetes Under Free-Living Conditions: Results of a Single-Arm 1-Month Experience Compared With a Previously Reported Feasibility Study of Evening and Night at Home. Diabetes Care. 2016;39(7):1151-1160. doi:10.2337/dc16-0008
3. Baron DN, Broughton PM, Cohen M, Lansley TS, Lewis SM, Shinton NK. The use of SI units in reporting results obtained in hospital laboratories. Ann Clin Biochem. 1974;11(5):194-202. doi:10.1177/000456327401100154

**The sample size calculation should be revised and better explained.**

Dear Editor, based on your and the reviewer’s suggestions, we understand that the main clarity issue regarding the sample size calculation stems from the use of 62% (0.62) in our sample size calculation. We revised the section to provide a better explanation why this proportion was used.

We indicated that the sample size was determined using a single population proportion formula which is commonly used in population based survey studies.

The 62% used in the calculation represents the prevalence of herbal medicine use among type 2 diabetes patients from a previous study in a similar setting. This study was conducted as one of the themes of a broader project proposed to assess the impacts of herbal medicine use on clinical outcomes and health-related quality of life among type 2 diabetes patients. In fact, herbal medicine use is considered as one of the factors associated with both glycemic control and health-related quality of life in this specific study. At the time of methodology development, we decided to base the sample size calculation on the prevalence of herbal medicine use reported in existing literature. Because the overall project also aimed to assess herbal medicine use. Since this variable was relevant across all themes of the broader study, including this specific manuscript, we considered it appropriate and applicable parameter for calculating the sample size for the project.

That is why we put this approach in the sample size calculation and we explained it subsequent to the formula. We have already attempted to explain this concept in the previous versions of the manuscript, and in this revision, we have worked on it to refine the clarity in brief and concise manner (on page 6 in the revised manuscript).

In summary:

- We used single population formula involving 62% and we get 362 initial sample size.
- We added 15% contingency for potential nonresponse and the final sample size considered for the study was 416.
- 386 were included in the final analysis (specific for this manuscript) because some participants were excluded essentially for data inconsistency problem.

With best regards,

Assefa Belay Asrie (Corresponding author)
