# Supplementary material for: Assessment of glycemic control, health-related quality of life, and associated factors in type 2 diabetic patients attending a comprehensive specialized hospital in Northwest Ethiopia
Source: PLoS One. 2025 Sep 18;20(9):e0332628. doi: 10.1371/journal.pone.0332628 (PMC12445461; doi:10.1371/journal.pone.0332628)
Supplement: Attachment — Submitted filename: Response to editor and reviewer comments.docx [file pone.0332628.s003.docx]

**Response to Editor and Reviewer Comments**

**Manuscript Title:** Glycemic control and health-related quality of life and associated factors in type 2 diabetic patients attending a comprehensive specialized hospital in northwest Ethiopia

**Manuscript Submission Number:** PONE-D-25-24519

**Journal:** PLOS ONE

**Response to the Editor**

Dear Editor,

We extend our heartfelt gratitude to you for handling the publication process of our manuscript. We strongly believe that your skillful management and suggestions are instrumental in ensuring the smooth progress and successful completion of this endeavor.

Changes made or the new texts included in the revision process are highlighted in blue font color. Deletions are shown by red colored texts with Strikethrough line, ~~example~~.

Journal Requirements:

When submitting your revision, we need you to address these additional requirements.

1. Please ensure that your manuscript meets PLOS ONE's style requirements, including those for file naming.

We ensure that the manuscript meets the journal’s style requirements.

2. In the online submission form you indicate that your data is not available for proprietary reasons and have provided a contact point for accessing this data. Please note that your current contact point is a co-author on this manuscript. According to our Data Policy, the contact point must not be an author on the manuscript and must be an institutional contact, ideally not an individual. Please revise your data statement to a non-author institutional point of contact, such as a data access or ethics committee, and send this to us via return email. Please also include contact information for the third party organization, and please include the full citation of where the data can be found.

Thank you dear Editor for your message. We would like to notify that our data is not associated with any proprietary restrictions. The statement provided in the online submission form regarding proprietary reasons was likely an unintentional error. Importantly, no such restriction was stated in the ‘Data availability statement’ of the originally submitted manuscript.

The full dataset is available in excel format and can be shared without restriction. It is currently held by the corresponding author and submitted as Supporting Information alongside the revised manuscript. The data availability statement has been revised accordingly.

3. If the reviewer comments include a recommendation to cite specific previously published works, please review and evaluate these publications to determine whether they are relevant and should be cited. There is no requirement to cite these works unless the editor has indicated otherwise.

The reviewer did not suggest any specific references for citation.

4. Please review your reference list to ensure that it is complete and correct. If you have cited papers that have been retracted, please include the rationale for doing so in the manuscript text, or remove these references and replace them with relevant current references. Any changes to the reference list should be mentioned in the rebuttal letter that accompanies your revised manuscript. If you need to cite a retracted article, indicate the article’s retracted status in the References list and also include a citation and full reference for the retraction notice.

We have thoroughly reviewed the reference list to ensure it is complete and accurate. Additionally, we confirm that no retracted papers have been cited.

Additional Editor Comments (if provided):

The paper requires some changes as indicated by reviewer 1. These requests need to be addressed in full and in an appropriate way.

Thank you, dear Editor! We have addressed all the requests raised by Reviewer 1. A detailed point-by-point response is provided in the response letter, and the corresponding revisions are highlighted in the manuscript.

**Response to Reviewer #1**

Dear reviewer,

We are grateful for your time and dedication in reviewing our manuscript. Your insightful comments are greatly appreciated and have been used to help improve the quality of the manuscript. We have addressed the comments as follows.

Changes made or newly added text during the revision process are highlighted in blue font color. Deletions are shown by red colored texts with Strikethrough line, ~~example~~.

Reviewer's Responses to Questions

**Comments to the Author**

1. Is the manuscript technically sound, and do the data support the conclusions?

Reviewer #1: Yes

Thank you!

2. Has the statistical analysis been performed appropriately and rigorously?

Reviewer #1: Yes

Thank you!

3. Have the authors made all data underlying the findings in their manuscript fully available?

Reviewer #1: Yes

Thank you!

4. Is the manuscript presented in an intelligible fashion and written in standard English?

Reviewer #1: Yes

Thank you!

5. Review Comments to the Author

Reviewer #1: This paper describes a cross-sectional cohort study of patients with type 2 diabetes attending a specialized hospital service in North West Ethiopia, and examines the relationship between a binary diabetes control category based on the average of three fasting blood glucose readings and health related quality of life. Overall the paper is technically sound and the findings are important in the context of a dearth of information from this region. The introduction is clear. The methods are fine, though it would be good to use SI units for the glucose measures. The sample size calculation seems convoluted – is this not just a convenience sample of patients, and if not, where did the figure of 62% come from? The results are clearly laid out and interpreted well in the discussion. Two minor points are that there is a typo in the title and the conclusions in the abstract are not necessarily justified by the findings described here.

Dear reviewer, we sincerely thank you again for your valuable feedback provided. We acknowledge the recognition of the technical soundness, clarity of the introduction, fineness of the methods, and clarity of the results and proper interpretation in the discussion. Moreover, we greatly acknowledge your recognition of the importance of the findings, in the context of limited information from the study region. We have addressed your comments as follows.

**It would be good to use SI units for the glucose measures.**

In accordance with this suggestion, we have revised the units for blood glucose measures in the manuscript to be presented in SI units (mmol/L). The following relation was used to change mg/dl values to mmol/L.

$$\frac{mg/dl}{18}=mmol/L$$

**Clarification of the use of 62% in the sample size calculation**

Thank you dear reviewer for your concern. We would like to clarify that this study is one of the themes of a broader project investigating the impact of herbal medicine use on clinical outcomes, including on blood glucose levels, and health-related quality of life among patients with type 2 diabetes. The sample size was, in fact, calculated using a single population proportion formula rather than through a convenience sampling method. The 62% figure used in the sample size calculation was based on the prevalence of herbal medicine use among type 2 diabetic patients, as reported in a previous study conducted in a similar setting. This proportion was chosen as the most appropriate for determining the required sample size for the project. We have already tried to state in the original submission why the figure (62%) was used in sample size calculation (with reference citation), in the methods section under subtitle “**Sample size and sampling technique”** just below the calculation of the sample size, page 5-6.

**A typo in the title**

Thank you for pointing out the typo in the title. We have corrected it, and the title is modified to: 'Assessment of glycemic control, health-related quality of life, and associated factors in type 2 diabetic patients attending a comprehensive specialized hospital in Northwest Ethiopia.' Additional changes made (other than typo correction) are intended to enhance the clarity of the title.

**The conclusions in the abstract are not necessarily justified by the findings described here.**

Upon consideration of this comment, we feel that there are statements which are not directly justifiable by the findings. With this note, we have deleted the statements. Please also note that we have made additional changes to this section based on the main results and with attention to enhancing clarity.

We have also made some changes to the conclusion of the main text (on page 26).

With sincere regards!

Assefa Belay Asrie (Corresponding author)
